# Protective Effects of *Alternanthera sessilis* Ethanolic Extract against TNF-*α* or H_2_O_2_-Induced Endothelial Activation in Human Aortic Endothelial Cells

**DOI:** 10.1155/2022/8738435

**Published:** 2022-02-24

**Authors:** Nur Nadia Mohd Razali, Soek Sin Teh, Siau Hui Mah, Yoke Keong Yong, Chin Theng Ng, Yang Mooi Lim, Lai Yen Fong

**Affiliations:** ^1^Department of Pre-Clinical Sciences, Faculty of Medicine and Health Sciences, Universiti Tunku Abdul Rahman, Kajang 43000, Selangor, Malaysia; ^2^Engineering and Processing Division, Malaysia Palm Oil Board, Kajang 43000, Selangor, Malaysia; ^3^School of Biosciences, Faculty of Health and Medical Sciences, Taylor's University, Subang Jaya 47500, Selangor, Malaysia; ^4^Department of Human Anatomy, Faculty of Medicine and Health Sciences, Universiti Putra Malaysia, Serdang 43400, Selangor, Malaysia; ^5^Unit of Physiology, Faculty of Medicine, AIMST University, Bedong 08100, Kedah, Malaysia

## Abstract

Activation of the endothelium has been shown to contribute to the early stage of vascular diseases such as atherosclerosis and hypertension. In endothelial activation, excess reactive oxygen species (ROS) production and increased expression of cell adhesion molecules cause an increase in vascular permeability. *Alternanthera sessilis* (L.) R. Br. is an edible traditional herbal plant, which has previously been shown to possess antioxidant and anti-inflammatory effects. However, the effect of *A. sessilis* on the activation of human aortic endothelial cells (HAECs) remains unknown. This study aimed to investigate the effects of *A. sessilis* on endothelial permeability, vascular cell adhesion-1 (VCAM-1) expression, production of ROS and hydrogen peroxide (H_2_O_2_), and superoxide dismutase (SOD) and catalase (CAT) activities. The viability of HAECs was first determined using the MTT viability assay. The effect of *A. sessilis* on endothelial permeability was examined using the FITC-dextran permeability assay. Besides, enzyme-linked immunosorbent assay (ELISA) was done to assess soluble VCAM-1 (sVCAM-1) expression. The production of ROS and H_2_O_2_ was studied using 2′,7′-dichlorodihydrofluorescein diacetate (H_2_-DCFDA) and Amplex Red fluorescent dyes, respectively. SOD and CAT activities were also measured using commercial kits. Our results showed that 25–200 *μ*g/mL of *A. sessilis* ethanolic extract did not cause significant death in HAECs. *A. sessilis* at 200 *μ*g/mL significantly inhibited TNF-*α*-induced hyperpermeability of HAECs. However, *A. sessilis* did not reduce increased VCAM-1 expression induced by TNF-*α*. *A. sessilis* also significantly reduced TNF-*α*-induced increased ROS production, but not H_2_O_2_ production. Furthermore, 100 *μ*M of H_2_O_2_ decreased both SOD and CAT activities in HAECs at 2 h. *A. sessilis* ethanolic extract dramatically increased both reduced SOD and CAT activities caused by H_2_O_2_. The liquid chromatography-mass spectrometry (LC-MS) analysis of *A. sessilis* ethanolic extract demonstrated the presence of arachidonic acid, azadirachtin, astaxanthin, flavanole base + 3O, 2Prenyl, and vicenin 2, while the gas chromatography-mass spectrometry (GC-MS) analysis showed that the extract contains 1,3,5-dihydroxy-6-methyl-2,3-dihydro-4H-pyran-4-one, 3-deoxy-d-mannoic lactone, 4-pyrrolidinobenzaldehyde, and *n*-hexadecanoic acid. In conclusion, our findings suggest that *A. sessilis* ethanolic extract protects against endothelial hyperpermeability and oxidative stress elicited by pro-inflammatory or prooxidant stimulus. This study reveals a therapeutic potential of *A. sessilis* in preventing endothelial activation, which is a key event in early atherosclerosis.

## 1. Introduction

The inner wall of blood vessels is lined by the endothelium, which functions as a semipermeable barrier that regulates permeability, inflammatory responses, and hemostasis [1]. Pro-inflammatory mediators such as tumor necrosis factor-alpha (TNF-*α*) have evidently been shown to induce endothelial activation, which is a key early event implicated in chronic inflammatory diseases such as atherosclerosis [2]. In endothelial activation, TNF-*α* and interleukins trigger vascular cell adhesion molecule 1 (VCAM-1) to be highly expressed on endothelial cells and stimulate increased endothelial permeability, thereby enhancing leukocyte attachment and migration to the site of inflammation [3]. Previous studies have highlighted the role of VCAM-1, as well as oxidative stress, in mediating endothelial activation [4]. Oxidative stress occurs when there is an imbalance between the release of prooxidant molecules such as reactive oxygen species (ROS) and hydrogen peroxide (H_2_O_2_), and the activity of antioxidant enzymes such as catalase (CAT) and superoxide dismutase (SOD). An overproduction of oxidants, which exceeds the ability of the antioxidant system to scavenge them, causes oxidative stress. The increased expression of adhesion molecules and oxidative stress in the activated endothelium initiate multiple signaling cascades that impair the endothelial barrier, resulting in increased endothelial permeability [5].

The current treatment for atherosclerosis focuses on the use of statins including simvastatin and atorvastatin, which act through the lipid-lowering approach. However, apart from adverse effects caused by statins, there is also evidence of relapses [6]. Thus, other alternative approaches such as targeting endothelial activation might be useful in the prevention and treatment of atherosclerosis. *Alternanthera sessilis* (L.) R. Br., popularly known as “sessile joy weed” or “dwarf copperleaf,” is a traditional herbal plant commonly found in Southeast Asia and other Asian countries such as Malaysia, Indonesia, India, and Pakistan [7]. The plant, belonging to the Amaranthaceae family, is used traditionally to treat a variety of conditions such as asthma, diabetes, wounds, diarrhea, and bronchitis [8, 9]. In Malaysia, *A. sessilis* is one of the commonly consumed *ulam* (salad), which is usually eaten raw or boiled with rice [10].

Previous *in vitro* studies showed that *A. sessilis* possesses a number of pharmacological activities such as antioxidant, anti-inflammatory, analgesic, and wound healing effects [11–13]. In RAW 264.7 cells, *A. sessilis* exhibited anti-inflammatory effect by inhibiting the production of pro-inflammatory mediators such as TNF-*α*, prostaglandin E2, interleukin-6, and interleukin-1*β* triggered by lipopolysaccharide [12]. Besides, a few *in vivo* studies also demonstrated hepatoprotective, antioxidant, analgesic, and antihyperglycemic effects of *A. sessilis* [14, 15]. *A. sessilis* was also demonstrated to suppress serum cholesterol and bilirubin levels, as well as lipid peroxidation in carbon tetrachloride (CCl_4_)-induced Wistar albino rats [7]. Activities of antioxidant enzymes such as CAT and glutathione peroxidase were also increased in the Wistar albino rat pretreated with *A. sessilis* methanolic extract. To date, no study has highlighted the protective effect of A*. sessilis* against barrier dysfunction and oxidative stress in the endothelium. Thus, this study aimed to investigate the effect of *A. sessilis* ethanolic extract in endothelial activation induced by TNF-*α* or H_2_O_2_ by examining endothelial permeability, VCAM-1 expression, production of ROS and H_2_O_2_, and activities of SOD and CAT. Identification of compounds in *A. sessilis* extract was also done using liquid chromatography-mass spectrometry (LC-MS) and gas chromatography-mass spectrometry (GC-MS).

## 2. Materials

Human recombinant TNF-*α* was purchased from PeproTech (NJ, USA). H_2_O_2_ was purchased from Merck. Simvastatin, 2′,7′-dichlorodihydrofluorescein diacetate (H_2_-DCFDA), fluorescein isothiocyanate (FITC)-dextran, dexamethasone, and N-acetyl cysteine (NAC) were purchased from Sigma-Aldrich (MO, USA). 10X trypsin-EDTA was purchased from Biowest (Nuaille, France). 3-(4,5-dimethylthiazol-2-yl)-2,5-diphenyltetrazolium bromide (MTT), dimethyl sulfoxide (DMSO), and phosphate-buffered saline (PBS) were obtained from Oxoid (UK).

## 3. Methods

### 3.1. Preparation of *A. sessilis* Ethanolic Extract

*A. sessilis* whole plant was collected from the herb garden of Persatuan Memperbaiki Akhlak Che Ru, Endau, Johor, Malaysia (2°38′56.8″N 103°37′49.7″E), in July 2014. A sample of the plant with voucher specimen number RG5040 was deposited at the Department of Biology, Faculty of Science, Universiti Putra Malaysia, and the plant was verified by Dr. Rusea Go. Approximately 104.4 g of air-dried *A. sessilis* was ground to fine powder and extracted in a Soxhlet apparatus with ethanol for 4 h [16]. The extract was evaporated to dryness under vacuum to give 31.3 g of ethanolic extract. To prepare a stock solution of *A. sessilis* ethanolic extract, the extract was dissolved in endothelial basal media to a concentration of 5 mg/mL. The stock was filter-sterilized through a 0.2 *μ*m polyethersulfone (PES) membrane filter before being stored at 4°C for a maximum of 3 months. The working solutions were freshly prepared using endothelial cell media on the day of usage. Any leftover was discarded.

### 3.2. Cell Culture

Primary human aortic endothelial cells (HAECs) were purchased from American Type Culture Collection (VA, USA). HAECs were cultured in endothelial cell media supplemented with 5% (v/v) fetal bovine serum (FBS), 100 U/mL penicillin/streptomycin, and 1% endothelial cell growth factor (ScienCell, CA, USA) and were grown in T-25 culture flasks at a starting density of 2.5 × 10^3^ cells/cm^2^. The cells were maintained at 37°C in a 5% CO_2_ incubator. The media were changed the next day after thawing and subsequently every two days until the cells reached a confluency of about 70%. Then, the media were changed every day until the cells were 80–90% confluent. The cells were subcultured until the desired passage. Cells at passages 3 to 5 were used for assays.

### 3.3. MTT Viability Assay

The cell viability was determined according to the procedures described previously [17]. HAECs were seeded onto 96-well plates at a density of 1 × 10^4^ cells/well overnight. The medium was removed, and the cells were then incubated with 25, 50, 100, 200, 400, and 800 *μ*g/mL of *A. sessilis* ethanolic extract for 24 h.

Then, 10 *μ*l of 5 mg/mL MTT (Sigma-Aldrich, MO, USA) in PBS was added to each well, and the cells were incubated for another 4 h. Lastly, all solution was removed from the well, and 100 *μ*L of dimethyl sulfoxide (DMSO) was added to each well. The absorbance was read using a microplate reader (Infinite M200pro, Tecan, Switzerland) at 450 nm. The cell viability was expressed as a percentage of untreated control.

### 3.4. FITC-Dextran Permeability Assay

This assay was performed according to the procedure previously described with some modifications [18]. Cell culture inserts with a pore size of 1.0 *μ*m (Falcon, USA) were coupled with 24-well companion plates. 2 × 10^5^ cells were seeded on each cell culture insert precoated with 1.5 mg/mL of type 1 rat tail collagen (BD Biosiences, USA). Each bottom chamber was filled with 500 *μ*L of endothelial cell media. The cells were grown for 3 to 4 days until a monolayer was formed. After the cells formed a monolayer, the cells were then treated with *A. sessilis* ethanolic extract (25–200 *μ*g/mL) or simvastatin (2 *μ*M) for 24 h. Then, the cells were induced with 10 ng/mL of TNF-*α* for 6 h. Following treatment, the media in the insert and in the bottom well were removed. The bottom well was filled with 500 *μ*Lof endothelial basal media, while 150 *μ*L of 0.04 mg/mL FITC-dextran (2000 kDa) was added to the insert and incubated at room temperature. After 20 mins, the permeation of FITC-dextran was stopped by removing the culture insert from the well. The fluorescence intensity was then measured using a fluorescent microplate reader (Infinite M200pro, Tecan, Switzerland) at an excitation wavelength of 485 nm and an emission wavelength of 530 nm.

### 3.5. Measurement of Soluble VCAM-1 (sVCAM-1) Production

The assay was performed using DuoSet VCAM-1/CD106 enzyme-linked immunosorbent assay (ELISA) and ancillary reagent kit (R&D System, Minnesota, USA) according to the manufacturer's protocol. HAECs were seeded in 24-well plates at a density of 2 × 10^5^ cells/well overnight. The cells were then treated with *A. sessilis* ethanolic extract at 25, 50, 100, and 200 *μ*g/mL, 2 *μ*M of simvastatin, or 10 mM of NAC for 24 h. Then, the cells were induced with 10 ng/mL TNF-*α* for 6 h. The supernatant was collected and was centrifuged at 1500 rpm for 10 mins at 4°C. Then, the supernatant was collected in new tubes and stored at −80°C if not assayed on the same day. To perform the assay, samples were first diluted at a dilution factor of 2 and were added to mouse antihuman VCAM-1 capture antibody-coated ELISA plate. Then, biotinylated sheep antihuman VCAM-1 detection antibody was added to each well, and this was followed by the addition of streptavidin-horseradish peroxidase. H_2_O_2_ and tetramethylbenzidine mixture was added to each well, and the reaction was stopped by the addition of 2 N H_2_SO_4_. The absorbance was read at 450 nm and corrected at 540 nm using a microplate reader (Infinite M200pro, Tecan, Switzerland).

### 3.6. Intracellular Reactive Oxygen Species (ROS) Quantitative Assay

This assay was done according to the method of Ganji et al. with some modifications [19]. HAECs were seeded in 96-well plates at a density of 1 × 10^4^ cells/well, and the plate was incubated overnight. To optimize the duration of TNF-*α* required to induce maximal ROS level production, the medium in each well was removed and the cells were incubated with 10 *μ*M of H_2_-DCFDA (Sigma-Aldrich, MO, USA) for 30 mins. After that, 10 ng/mL of TNF-*α* (PeproTech, NJ, USA) was added to the well and the plate was further incubated for 30 mins, 1 h, 2 h, 4 h, 6 h, and 24 h. The fluorescence intensity was measured at excitation and emission wavelengths of 480 nm and 570 nm, respectively, using a fluorescent microplate reader (Infinite M200pro, Tecan, Switzerland). The results were expressed as a percentage of control.

To study the effect of *A. sessilis* on TNF-*α*-induced ROS production, the cells were pretreated with 25, 50, 100, and 200 *μ*g/mL of *A. sessilis* ethanolic extract for 24 h before the addition of H_2_-DCFDA fluorescent dye. Then, the cells were induced with 10 ng/mL of TNF-*α* for 4 h.

### 3.7. Hydrogen Peroxide (H_2_O_2_) Assay

The assay was performed using Amplex Red H_2_O_2_/peroxidase assay kits (Invitrogen, USA) according to the manufacturer's protocol. HAECs were cultured overnight in 6-well plates at a density of 3 × 10^5^ cells/well. First, the TNF-*α* concentration and induction time required to increase H_2_O_2_ level were optimized. The cells were induced with 10, 20, 100, and 200 ng/mL of TNF-*α* for 30 mins, 1 h, 2 h, 4 h, and 6 h. The supernatant was collected and was immediately assayed using the kit. The samples were mixed with 0.1 mM of Amplex Red and 0.2 U/mL of horseradish peroxidase solution diluted in 1X reaction buffer. After 30 mins, fluorescence intensities were measured using a microplate reader at excitation/emission wavelengths of 540/590 nm (Infinite M200pro, Tecan, Switzerland). To study the effects of *A. sessilis* on H_2_O_2_ production, the cells were treated with 25, 50, 100, and 200 *μ*g/mL of *A. sessilis* or 2 *μ*M of simvastatin for 24 h before an induction with 20 ng/mL of TNF-*α* for 1 h.

### 3.8. Measurement of Superoxide Dismutase (SOD) Activity

The SOD activity was detected using SOD Assay Kits (Cayman, USA). Briefly, HAECs were seeded onto 12-well plates at a density of 2 × 10^5^ cells/well. To optimize the concentration and induction time of H_2_O_2_, the cells were induced with H_2_O_2_ at concentrations of 50, 100, 200, and 400 *μ*M for 30 mins, 1 h, and 2 h. To assess the effects of *A. sessilis* on SOD production, the cells were treated with 25, 50, 100, and 200 *μ*g/mL of* A. sessilis* ethanolic extract or 2 *μ*M of simvastatin or 10 *μ*M of NAC or 10 *μ*M of dexamethasone for 24 h, followed by an induction with 100 *μ*M of H_2_O_2_ for 2 h. After the indicated treatment, the medium was removed from each well and replaced with ice-cold buffer. The cells were harvested using rubber cell scrapers, and the cell lysates were centrifuged at 1500 ×g for 5 mins at 4°C. The supernatant was collected and stored at −80°C. To measure SOD activity, the samples were first added to tetrazolium salt radical detector. Then, xanthine oxidase was added to initiate the reaction. After 30 mins, the absorbance was read at 440 nm using a microplate reader (Infinite M200pro, Tecan, Switzerland).

### 3.9. Measurement of Catalase (CAT) Activity

CAT activity was detected using CAT Assay Kits (Cayman, USA) according to the manufacturer's protocol. Briefly, HAECs were seeded onto 6-well plates at a density of 3 × 10^5^ cells/well. In optimization experiments, the cells were induced with 50, 100, 200, and 500 *μ*M of H_2_O_2_ for 30 mins, 1 h, and 2 h. To study the effects of *A. sessilis* on CAT activity, the cells were treated with 25, 50, 100, and 200 *μ*g/mL of*A. sessilis* or 2 *μ*M of simvastatin for 24 h, followed by induction with 100 *μ*M of H_2_O_2_ for 2 h.

The cells were harvested in ice-cold buffer and were centrifuged at 10,000 ×g for 15 mins at 4°C. The supernatant was collected and used for the assay. Briefly, samples, methanol, and assay buffer were added to 96-well assay plates. Then, 20 *μ*L of H_2_O_2_ was added to each well to initiate the reaction, which was stopped with 30 *μ*L of potassium hydroxide after 20 mins. Catalase potassium periodate was then added at a volume of 10 *μ*L, and the absorbance was read at 540 nm.

### 3.10. Liquid Chromatography-Mass Spectrometry (LC-MS)

*A. sessilis* ethanolic extract was separated using Thermo Scientific C18 Column (3 × 150 mm, 3 *μ*m particle size; Acclaim^TM^ Polar Advantage II, Thermo Scientific, USA) with an UltiMate 3000 UHPLC System (Dionex, Thermo Fisher Scientific, USA). Gradient elution was run at this setting: 0.4 mL/min and 40°C using water and 0.1% formic acid (A) and 100% acetonitrile (B) with a total run time of 22 mins. The sample was injected at a volume of 1 *μ*L. The gradient began at 5% B (0–3 mins); 80% B (3–10 mins); 80% B (10–15 mins); and 5% B (15–22 mins). High-resolution mass spectrometry was performed with micrOTOF-QIII (Bruker Daltonics GmbH, Germany) using an ESI-positive ionization and the following conditions: capillary voltage: 4500 V; nebulizer pressure: 1.2 bar; and drying gas: 8 L/min at 200°C. The mass range was set at 50–1000 m/z. The mass data of molecular ions were then processed and analyzed using Compass Data Analysis 4.1 software (Bruker Daltonics GmbH, Germany).

### 3.11. Gas Chromatography-Mass Spectrometry (GC-MS)

*A. sessilis* ethanolic extract was analyzed using GC-MS (Agilent J&W, USA) equipped with GC column HP-5MS (30 m × 0.25 mm × 0.25 *μ*m). The temperature of the column was first set to 60°C, was gradually increased to 250°C at a rate of 15°C/min, and was then kept constant for 8 mins. The injector temperature was fixed at 200°C (split mode with ratio 10 : 1, injection volume 1 *μ*L) with a total run time of 20 mins. The mass spectra were obtained between the range m/z 50 and 550 and the electron ionization at 70 eV. The mass spectra were then compared with the NIST17 library data to identify chromatograms of the sample.

### 3.12. Statistical Analysis

All experiments were performed in triplicates and repeated for at least three times. The results were analyzed and presented as the means of readings and standard error of means (mean ± S.E.M). Statistical analysis was performed using one-way analysis of variance (ANOVA) and Dunnett's test in GraphPad Prism 7 (CA, USA). The statistical difference of *P* < 0.05 was considered as significant.

## 4. Results

### 4.1. *A. sessilis* Does Not Affect Viability of HAECs at 6.25–200 *μ*g/mL

**Figure 1:**
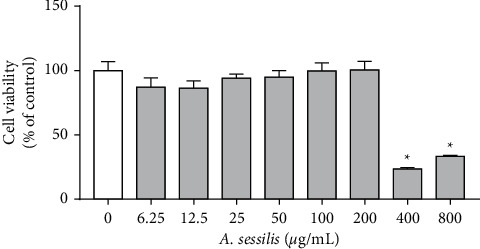
Effect of *A. sessilis* ethanolic extract on HAEC viability. HAECs were treated with various concentrations of *A. sessilis* ethanolic extract (6.25–800 *μ*g/mL) for 24 h. The percentage of cell viability was determined using the MTT assay. Data are presented as the mean ± SEM of three independent experiments. ^∗^*P* < 0.05 as compared to the untreated control.

HAECs were treated with 6.25–800 *μ*g/mL of *A. sessilis* ethanolic extract for 24 h, and the MTT assay was used to evaluate whether *A. sessilis* ethanolic extract would affect the cell viability. *A. sessilis* ethanolic extract, at 400 and 800 *μ*g/mL, was found to cause significant cell death with mean cell viabilities of 23.74 ± 0.83 and 33.42 ± 0.81% of control, respectively (Figure 1) (*P* < 0.05), whereas 6.25–200 *μ*g/mL of*A. sessilis* ethanolic extract did not affect the viability of HAECs. Therefore, the four highest concentrations (25, 50, 100, and 200 *μ*g/mL), which did not cause significant cell death, were used in subsequent experiments.

### 4.2. *A. sessilis* Suppresses Increased Endothelial Permeability Caused by TNF-*α*

**Figure 2:**
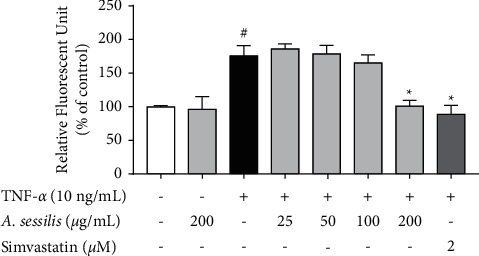
Effect of *A. sessilis* ethanolic extract on TNF-*α*-induced hyperpermeability of HAECs. HAECs cultured on collagen-coated transwell inserts were pretreated with various concentrations of *A. sessilis* (25–200 *μ*g/mL) or simvastatin (2 *μ*M) for 24 h followed by stimulation with TNF-*α* (10 ng/mL) for 6 h. At the end of the experiment, the media in the bottom well was collected, and fluorescence intensities of the bottom well were measured. Data are presented as the mean ± SEM of three independent experiments, where each was performed in triplicates. ^#^*P* < 0.05 as compared to the unstimulated control. ^∗^*P* < 0.05 as compared to the TNF-*α*-treated group.

*In vitro* vascular permeability assay is an assay used to assess the permeability of endothelial cells by measuring the passage of fluorescent probes across a cell monolayer grown on collagen-coated inserts. The effect of *A. sessilis* ethanolic extract on endothelial cell permeability was studied. As shown in Figure 2, 10 ng/mL of TNF-*α* significantly increased the permeability of HAEC monolayer to 175.8 ± 15.03% of control (*P* < 0.05) (Figure 2) at 6 h. Pretreatment with 200 *μ*g/mL of* A. sessilis* ethanolic extract for 24 h significantly reduced the increased permeability stimulated by TNF-*α* to a level, which was comparable to the control group (101.0 ± 8.26% of control) (*P* < 0.05). Simvastatin, the positive control, administered at 2 *μ*M, also significantly decreased TNF-*α*-induced hyperpermeability to 88.64 ± 13.32% of control (*P* < 0.05). However, 25, 50, and 100 *μ*g/mL of *A. sessilis* ethanolic extract did not have a significant effect on the increased endothelial permeability. These results showed that *A. sessilis* ethanolic extract prevents the impairment of the endothelial barrier induced by TNF-*α*, which is indicated by the suppression of FITC-dextran permeability.

### 4.3. *A. sessilis* Does Not Inhibit TNF-*α*-Induced Release of sVCAM-1

**Figure 3:**
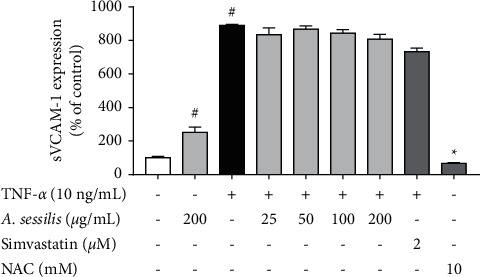
Effect of *A. sessilis* ethanolic extract on TNF-*α*-induced increased sVCAM-1 release in HAECs. HAECs were pretreated with various concentrations of *A. sessilis* (25–200 *μ*g/mL), simvastatin (2 *μ*M), or NAC (10 mM) for 24 h followed by stimulation with 10 ng/mL of TNF-*α* for 6 h. The supernatant was collected for assay using VCAM-1 ELISA kits. The absorbance was measured at 450 nm and corrected at 540 nm. ^#^*P* < 0.05 as compared to the unstimulated control. ^∗^*P* < 0.05 as compared to the TNF-*α*-treated group.

sVCAM-1 released in the cell culture supernatant was measured using commercial ELISA kits. The sVCAM-1 production was dramatically increased by 10 ng/mL of TNF-*α* to 890.2 ± 6.87% (*P* < 0.05), compared with unstimulated control (Figure 3). Pretreatment with *A. sessilis* ethanolic extract at all concentrations (25–200 *μ*g/mL) did not inhibit TNF-*α*-induced sVCAM-1 production. Surprisingly, treatment with 200 *μ*g/mL of*A. sessilis* ethanolic extract alone significantly increased VCAM-1 expression to 251.5 ± 31.01% (*P* < 0.05) in HAECs. The experiment was also validated with the use of NAC, which is a ROS inhibitor. 10 mM of NAC dramatically reduced sVCAM-1 expression induced by TNF-*α* to 67.17 ± 4.61% of control (*P* < 0.05). These data suggested that *A. sessilis* does not alter the secretion of sVCAM-1 caused by TNF-*α*.

### 4.4. *A. sessilis* Reduces TNF-*α*-Induced Increased Intracellular ROS Levels

Intracellular ROS production was measured using H_2_-DCFDA dye. This nonfluorescent dye penetrates cell membranes and is converted to a fluorescent molecule, 2′, 7'-dichlorofluorescein (DCF), in the presence of ROS. To determine at which time point TNF-*α* induces the highest ROS accumulation in HAECs, the cells were stimulated with 10 ng/mL of TNF-*α* for 30 mins, 1 h, 2 h, 4 h, 6 h, and 24 h prior to the measurement of ROS production. We showed that 10 ng/mL of TNF-*α* significantly increased intracellular ROS levels at 4 h (134 ± 2.27% of control), compared with unstimulated control (Supplementary Figure 1). Therefore, 4 h incubation time for TNF-*α* was used in the subsequent experiment to study the effect of *A. sessilis* ethanolic extract in increased intracellular ROS levels stimulated by TNF-*α*.

**Figure 4:**
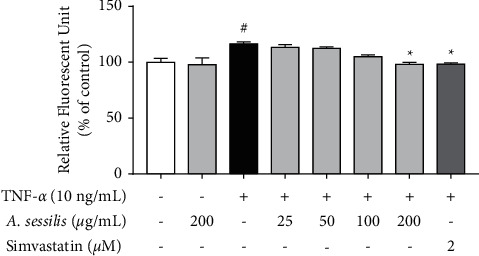
Effect of *A. sessilis* ethanolic extract on TNF-*α*-induced increased ROS levels in HAECs. HAECs were pretreated with various concentrations of *A. sessilis* (25–200 *μ*g/mL) or simvastatin (2 *μ*M) for 24 h prior to TNF-*α* (10 ng/mL) induction for 4 h. After staining the cells with H_2_-DCFDA for 30 mins, the relative fluorescence unit of each well was measured. Data are presented as the mean ± SEM of three independent experiments (*n* = 3). ^#^*P* < 0.05 as compared to the unstimulated control. ^∗^*P* < 0.05 as compared to the TNF-*α*-treated group.

In Figure 4, the results showed that TNF-*α* significantly increased intracellular ROS levels to 116.6 ± 1.67 of control (*P* < 0.05) at 4 h and this was inhibited by pretreatment of 200 *μ*g/mL of *A. sessilis* ethanolic extract (98.1 ± 1.84% of control) (*P* < 0.05). Consistent with the finding from the permeability assay, pretreatment with 25–100 *μ*g/mL of *A. sessilis* ethanolic extract did not alter TNF-*α*-induced ROS release in HAECs. In summary, *A sessilis* suppresses the release of intracellular ROS stimulated by TNF-*α*.

### 4.5. *A. sessilis* Fails to Reduce Extracellular H_2_O_2_ Production Induced by TNF-*α*

As the ROS assay did not indicate the type of ROS produced in cells, we then measured the release of H_2_O_2_, one of the most important ROS that causes endothelial activation, in cell culture supernatant using Amplex Red dye. The optimization data showed that 20 and 100 ng/mL of TNF-*α* significantly increased extracellular H_2_O_2_ levels at 1 h (138.6 ± 4.69% of control and 171.6 ± 10.66% of control, respectively) (Supplementary Figure 2). It has been reported that 100 ng/mL of TNF-*α* caused apoptosis of human lung endothelial cells [20]. To exclude the apoptotic effect of TNF-*α*, 20 ng/mL of TNF-*α* and 1 h incubation time were used in the subsequent experiment to measure the effect of *A. sessilis* ethanolic extract on TNF-*α*-stimulated H_2_O_2_ production.

**Figure 5:**
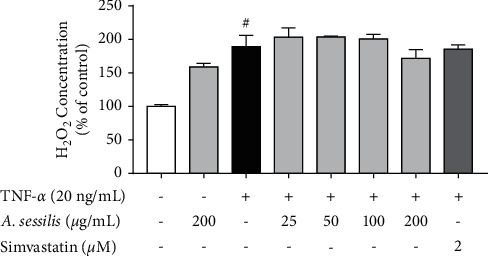
Effect of *A. sessilis* ethanolic extract on TNF-*α*-induced extracellular H_2_O_2_ production. HAECs were pretreated with various concentrations of *A. sessilis* (25–200 *μ*g/mL) or 2 *μ*M of simvastatin for 24 h followed by stimulation with 20 ng/mL of TNF-*α* for 1 h. The assay was performed immediately using H_2_O_2_ assay kits after the collection of cell culture supernatant. The results are presented as the mean ± SEM from three independent experiments (*n* = 3). ^#^*P* < 0.05 as compared to the unstimulated control.

In the subsequent experiment, HAECs induced with 20 ng/mL TNF-*α* for 1 h showed an increase in H_2_O_2_ production (189 ± 16.95% of control) (Figure 5). However, neither *A. sessilis* ethanolic extract nor simvastatin reduced the elevated H_2_O_2_ production induced by TNF-*α*.

### 4.6. *A. sessilis* Improves H_2_O_2_-Stimulated Reduced SOD Activity

Based on our preliminary study, TNF-*α* did not cause a significant decrease in SOD activity in HAECs at the concentration range of 10–200 ng/mL and the incubation period of 1–6 h (data not shown). Therefore, H_2_O_2_ was used to study SOD activity in HAECs. To identify the optimum induction period of H_2_O_2_, HAECs were treated with 50–400 *μ*M of H_2_O_2_ for 30 mins, 2 h, and 4 h. The data showed that 100 *μ*M of H_2_O_2_ significantly reduced SOD activity at 2 h (19.92 ± 7.1% of control), compared with unstimulated control (Supplementary Figure 3). HAECs were induced with 100 *μ*M of H_2_O_2_ for 2 h in the subsequent experiment, which measured the effect of *A. sessilis* ethanolic extract on H_2_O_2_-induced reduced SOD activity.

**Figure 6:**
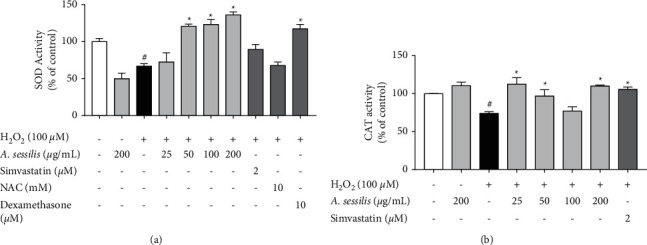
Effect of *A. sessilis* ethanolic extract on H_2_O_2_-induced reduced (a) SOD and (b) CAT activities. HAECs were pretreated with various concentrations of *A. sessilis* (25–200 *μ*g/mL), simvastatin (2 *μ*M), NAC (10 mM), or dexamethasone (10 *μ*M) for 24 h followed by stimulation with 100 *μ*M of H_2_O_2_ for 2 h. Cell lysates were collected, and the assay was performed immediately using SOD or CAT assay kits. The results are presented as the mean ± SEM from three independent experiments (*n* = 3). ^#^*P* < 0.05 as compared to the unstimulated control. ^∗^*P* < 0.05 as compared to the H_2_O_2_-induced group.

In the subsequent experiment, we demonstrated that 100 *μ*M of H_2_O_2_ significantly reduced SOD activity in HAECs to 66.78 ± 3.37 of control (*P* < 0.05) (Figure 6(a). Pretreatment of 50, 100, and 200 *μ*g/mL of *A. sessilis* ethanolic extract significantly increased H_2_O_2_-stimulated decreased SOD activity in a dose-dependent manner (120.7 ± 3.15%, 123.2 ± 6.67%, and 136.1 ± 4.01% of control, respectively). HAECs that were pretreated with 10 *μ*M of dexamethasone also showed an increase in SOD activity (117.4 ± 5.8% of control) (*P* < 0.05). Besides, 200 *μ*g/mL of *A. sessilis* ethanolic extract alone was also found to alter the activity of SOD.

### 4.7. *A. sessilis* Elevates Reduced CAT Activity Induced by H_2_O_2_

The optimization result showed that 100 *μ*M and 500 *μ*M of H_2_O_2_ significantly reduced CAT activity at 2 h, compared with unstimulated control (Supplementary Figure 4). The concentration of 100 *μ*M and 2 h incubation time were chosen to be used in the subsequent experiment. 100 *μ*M of H_2_O_2_ was chosen as 500 *μ*M of H_2_O_2_ has previously been demonstrated to affect cell viability [21].

As shown in Figure 6(b), 100 *μ*M of H_2_O_2_ significantly lowered CAT activity in HAECs to 74.08 ± 2.07 of control (*P* < 0.05). *A. sessilis* ethanolic extract, at 25, 50, and 200 *μ*g/mL, significantly elevated the lowered CAT activity caused by H_2_O_2_ to 112.5 ± 8.65%, 96.84 ± 8.46%, and 110.1 ± 1.28% of control, respectively. A non-dose-dependent effect was observed, though. Taken together, *A. sessilis* ethanolic extract ameliorates the reduced activities of antioxidant enzymes elicited by H_2_O_2._

### 4.8. Identification of Compounds through LC-MS Analysis

**Figure 7:**
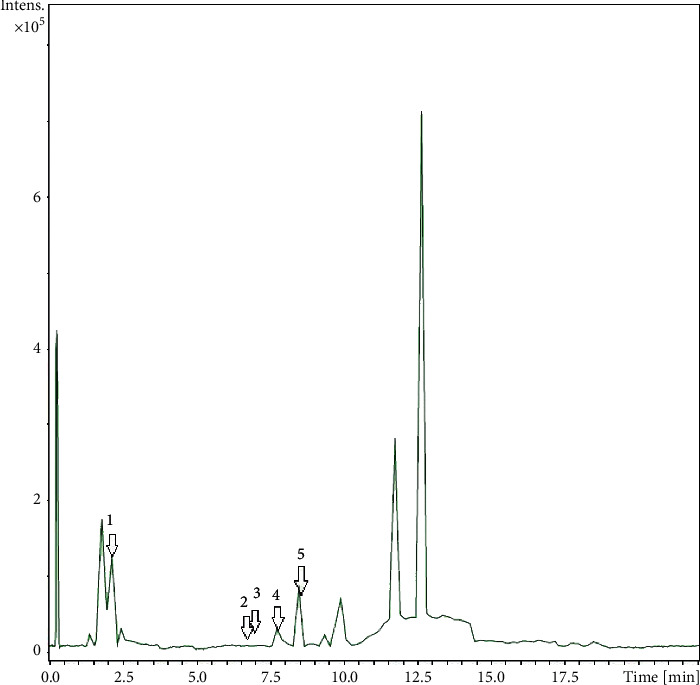
Liquid chromatogram of *A. sessilis* ethanolic extract.

LC-MS analysis on *A. sessilis* ethanolic extract chromatogram showed the presence of 81 peaks (Figure 7). Of the 81 peaks, five compounds were successfully identified using the MassBank library. The compounds identified were arachidonic acid, azadirachtin, astaxanthin, flavanole base + 3O, 2Prenyl, and vicenin 2 [22–24]. The retention time, m/z values, and molecular formula of the compounds are presented in Table 1. The MS/MS spectra of the compounds identified are shown in Supplementary Figures 5.

### 4.9. Identification of Compounds through GC-MS Analysis

**Figure 8:**
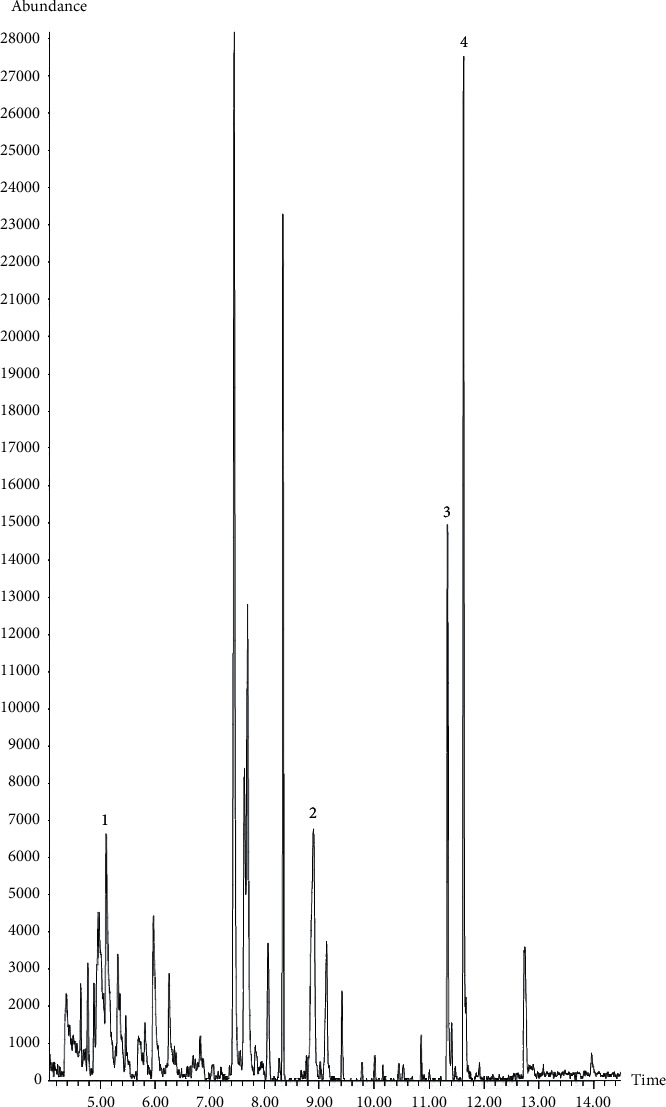
Gas chromatogram of *A. sessilis* ethanolic extract.

GC-MS analysis on *A. sessilis* ethanolic extract chromatogram showed the presence of eight prominent peaks (Figure 8). Of the eight peaks identified, four compounds were found to match with the library and the compounds identified were 1,3,5-dihydroxy-6-methyl-2,3-dihydro-4H-pyran-4-one, 3-deoxy-d-mannoic lactone, 4-pyrrolidinobenzaldehyde, and n-hexadecanoic acid. The retention time, percentage of peak area concentration, and the molecular formula of the compounds are presented in Table 2.

## 5. Discussion

*A. sessilis* extracts have been demonstrated to suppress inflammatory responses in endotoxin-induced macrophages and to scavenge free radicals [12, 13]. However, the effect of *A. sessilis* on pro-inflammatory mediator-stimulated endothelial activation, particularly in terms of endothelial permeability, oxidative stress, and adhesion molecule expressions, remains poorly understood. In this study, we explored the protective effects of *A. sessilis* ethanolic extract against endothelial activation induced by TNF-*α* or H_2_O_2_. The optimization data showed that TNF-*α* induces increased endothelial permeability, sVCAM-1 expression, and production of both ROS and H_2_O_2_, but the cytokine does not stimulate significant reductions in both CAT and SOD activities. Therefore, H_2_O_2_ was used as an inducer to decrease CAT and SOD activities in this study. We demonstrated that *A. sessilis* ethanolic extract inhibits both endothelial hyperpermeability and increased ROS production stimulated by TNF-*α* in HAECs. However, *A. sessilis* fails to abrogate both the increased sVCAM-1 and H_2_O_2_ secretions. Importantly, *A. sessilis* ethanolic extract also elevates the reduced SOD and CAT activities caused by H_2_O_2_.

Paracellular permeability is regulated by tight junctions and adherens junctions that connect adjacent endothelial cells together to maintain the endothelial barrier. In endothelial activation, remodeling of interendothelial junctions takes place, causing increased endothelial permeability. In a dose- and time-response study of TNF-*α*, the cytokine has been shown to induce endothelial hyperpermeability in HAECs [17]. The disruption of endothelial barrier by TNF-*α*, which is characterized by increased endothelial permeability, results either from the direct action of TNF-*α* upon endothelial cells, or through the indirect effect triggered by leukocyte recruitment and adherence [25]. The results in this study showed that *A. sessilis* ethanolic extract protects against TNF-*α*-induced increased endothelial permeability (Figure 2), suggesting a barrier protective effect of *A. sessilis.*

VCAM-1 is one of the major regulators of leukocyte adhesion. The binding of integrins with VCAM-1 causes the production of O_2_^−^ and the resulting increased oxidative stress in endothelial cells [26]. VCAM-1 is also implicated as a key mediator in atherosclerosis, where its expression was abundantly detected at atherosclerotic lesion sites [26]. TNF-*α* is a well-known positive regulator of VCAM-1 as it was demonstrated to upregulate VCAM-1 mRNA expression [27]. However, this study showed that *A. sessilis* does not reduce the expression of sVCAM-1 induced by TNF-*α* (Figure 3). Besides that, simvastatin also failed to reduce the increased sVCAM-1 expression, and therefore, NAC was used as a control drug in the assay. Previous data imply that the effect of simvastatin on the endothelium is greatly dependent on shear stress levels applied to endothelial cells [28]. A previous study also demonstrated that simvastatin enhances CAM expressions in TNF-*α*-treated HUVECs [29].

In vascular endothelial cells, increased oxidative stress contributes to the development of vascular diseases including atherosclerosis and hypertension. ROS are molecules that have one or more unpaired electrons in their orbital. Among all, ROS, H_2_O_2_, and O_2_^−^ are known to contribute the most as signaling molecules that initiate oxidative stress [30]. Previous studies showed that TNF-*α* triggers increased ROS production in vascular cells, which results in increased endothelial permeability [31]. H_2_O_2_ functions as an important intracellular signaling molecule that maintains vascular homeostasis at physiological concentrations. In contrast, the excessive production of H_2_O_2_ causes endothelial barrier dysfunction, rearrangement of actin cytoskeleton, and apoptosis [32]. Furthermore, the free radical scavenging activity of *A. sessilis* extracts has been demonstrated previously using cell-free assays such as the 2,2-diphenyl-1-picryl-hydrazyl-hydrate (DPPH) assay, the reducing power assay, and many other methods [13, 33]. However, these assays do not mimic the *in vivo* environment of vascular beds where ROS is produced by several intracellular enzymes including nicotinamide adenine dinucleotide phosphate (NADPH) oxidases, xanthine oxidases, and uncoupled endothelial nitric oxide synthases. Our results showed that *A. sessilis* reduces intracellular ROS production, but not the release of H_2_O_2_, induced by TNF-*α* in the endothelium. This implies that *A. sessilis* may inhibit the generation of ROS other than H_2_O_2_.

**Figure 9:**
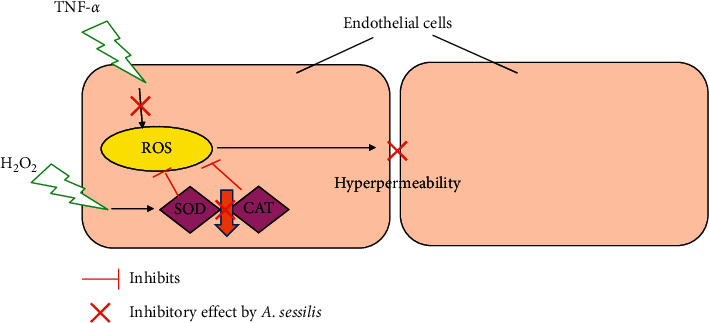
*A. sessilis* suppresses both TNF-*α*-induced endothelial hyperpermeability and increased ROS production. *A. sessilis* also prevents the reduction in SOD and CAT activities induced by H_2_O_2_. The endothelial barrier protective effect of *A. sessilis* might be attributed to its inhibitory effect on oxidative stress.

We then attempted to investigate whether the protective effect of *A. sessilis* against endothelial activation are also mediated by the enzymatic antioxidant mechanism. CAT and SOD are intracellular antioxidant enzymes that abrogate overproduction of ROS in cells. SOD converts O_2_^−^ to H_2_O_2_, which is less reactive, while CAT acts by reducing H_2_O_2_ into water and oxygen [34]. In this study, *A. sessilis* ethanolic extract successfully restored H_2_O_2_-induced decreased SOD and CAT activities (Figures 6(a) and 6(b)). Our results are in agreement with other previous findings. It has been reported that *A. sessilis* red ethyl acetate fraction dramatically increased pancreatic total SOD activity in diabetic rats [35]. Besides, dexamethasone was used as a positive control in the SOD assay of this study, as simvastatin and NAC did not prevent the reduced SOD activity induced by H_2_O_2_. Taken together, *A. sessilis* prevents oxidative stress by suppressing ROS generation and enhancing the antioxidant defense system in the endothelium. ROS has been shown to impair the endothelial barrier, which in turn causes increased vascular permeability. We suggest that the endothelial barrier protective effect of *A. sessilis* might be attributed to its inhibitory effect on oxidative stress (Figure 9).

Based on the LC-MS and GC-MS results, a total of five and four compounds were identified, respectively, in *A. sessilis* ethanolic crude extract, of which four were found to possess antioxidant and anti-inflammatory effects in *in vitro*, *in vivo,* and clinical studies (azadirachtin, astaxanthin, vicenin 2, and n-hexadecanoic acid) [36–39]. These compounds could be the active compounds, which are responsible for the positive effects of *A. sessilis* observed in this study.

There are some limitations in this study. Firstly, this study only evaluates the biological activity of *A. sessilis* crude extract and identifies compounds that are present in the crude extract. Bioassay-guided extraction, fractionation, and isolation of pure compounds should be done in the future to identify the active compound, which is responsible for the endothelial protective effect of *A. sessilis*. Secondly, the signaling pathway that underlies the endothelial protective effect of *A. sessilis* has not been identified in this study and further studies need to be done to address this. Lastly, this study provides only *in vitro* data, and therefore, *in vivo* studies also should be conducted in the future to better understand the *in vivo* effect of *A. sessilis* in suppressing vascular dysfunction and vascular oxidative stress.

## 6. Conclusion

In conclusion, this study shows that *A. sessilis* ethanolic extract protects against endothelial activation induced by TNF-*α* or H_2_O_2_. This is demonstrated by the protective effect of *A. sessilis* against TNF-*α*-induced increased endothelial permeability. In addition, *A. sessilis* also reduces intracellular ROS production and increases antioxidant enzyme activities (SOD and CAT). However, our data showed that *A. sessilis* does not inhibit sVCAM-1 secretion. An increase in endothelial permeability and oxidative stress are known to be indicators of endothelial activation, which plays an important role in the early stage of atherosclerosis. Therefore, this study suggests a new pharmacological activity of *A. sessilis* in preventing endothelial activation through inhibition of oxidative stress and endothelial hyperpermeability. Our data also support the medicinal use of this edible plant.

## Figures and Tables

**Table 1:** Compounds identified in *A. sessilis* crude extract using LC-MS analysis with the retention time, m/z value, and molecular formula.

Peak number	Compound	RT (min)	[M-H]^+^ (m/z)	Molecular formula
1	Arachidonic acid	2.1	305.1351	C_20_H_32_O_2_
2	Azadirachtin	6.9	720.0604	C_35_H_44_O_16_
3	Astaxanthin	7.0	597.179	C_40_H_52_O_4_
4	Flavanole base + 3O, 2Prenyl	7.7	409.3437	C_25_H_28_O_5_
5	Vicenin 2	8.5	595.167	C_27_H_30_O_15_

**Table 2:** Compounds identified in *A. sessilis* crude extract using GC-MS analysis with the retention time, peak area percentage, and molecular formula.

Peak number	Compound	RT (min)	Peak area (%)	Molecular formula
1	1,3,5-Dihydroxy-6-methyl-2,3-dihydro-4H-pyran-4-one	5.116	7.152	C_6_H_8_O_4_
2	3-Deoxy-d-mannoic lactone	8.898	5.877	C_6_H_10_O_5_
3	4-Pyrrolidinobenzaldehyde	11.336	7.069	C_11_H_13_NO
4	*n*-Hexadecanoic acid	11.629	10.69	C_16_H_32_O_2_

## Data Availability

The data of this study can be obtained from the corresponding author upon request.

## Supplementary Materials

Supplementary Materialsa
*Supplementary Figure 1.* Time response of TNF-*α* on ROS levels. HAECs were stimulated with 10 ng/mL of TNF-*α* for different durations (30 mins–24 h). After staining the cells with H_2_-DCFDA for 30 mins, the relative fluorescence unit of each well was measured. Data are presented as the mean ± SEM of three independent experiments (*n* = 3). ^∗^*P* < 0.005 compared with the unstimulated control. *Supplementary Figure 2.* Effect of TNF-*α* on extracellular H_2_O_2_ production in HAECs. HAECs were pretreated with various concentrations of TNF-*α* (10, 20, 100, or 200 ng/mL) for (a) 30 mins, (b) 1 h, (c) 2 h, (d) 4 h, and (e) 6 h. The supernatant was collected, and the assay was performed immediately using H_2_O_2_ assay kits. The results are presented as the mean ± SEM from three independent experiments (*n* = 3). ^#^*P* < 0.05 as compared to the unstimulated control. *Supplementary Figure 3.* Effect of H_2_O_2_ on SOD activity in HAECs. HAECs were pretreated with various concentrations of H_2_O_2_ (50, 100, 200, and 400 *μ*M) for (a) 30 mins, (b) 2 h, and (c) 4 h. Then, the cells were lysed in iced-cold buffer. The cell lysates were collected, and the assay was performed using SOD assay kits. Data are presented as the mean ± S.E.M of three independent experiments (*n* = 3). ^#^*P* < 0.05 as compared to the unstimulated control. *Supplementary Figure 4.* Effect of H_2_O_2_ on CAT activity in HAECs. HAECs were pretreated with various concentrations of H_2_O_2_ (50, 100, 200, and 500 *μ*M) for (a) 30 mins, (b) 2 h, and (c) 4 h. Cell lysates were collected, and CAT activity was measured using CAT assay kits. Data are presented as the mean ± SEM of three independent experiments (*n* = 3). #*P* < 0.05 as compared to the unstimulated control. Supplementary Figure 5: MS/MS spectrum of peaks 1–5.
