# Supplementary material for: Protective Effects of Alternanthera sessilis Ethanolic Extract against TNF-α or H2O2-Induced Endothelial Activation in Human Aortic Endothelial Cells
Source: Evid Based Complement Alternat Med. 2022 Feb 24;2022:8738435. doi: 10.1155/2022/8738435 (PMC8894009; doi:10.1155/2022/8738435)
Supplement: Supplementary Materialsa — Supplementary Figure 1. Time response of TNF-α on ROS levels. HAECs were stimulated with 10 ng/mL of TNF-α for different durations (30 mins–24 h). After staining the cells with H2-DCFDA for 30 mins, the relative fluorescence unit of each well was measured. Data are presented as the mean ± SEM of three independent experiments (n = 3). ∗P < 0.005 compared with the unstimulated control. Supplementary Figure 2. Effect of TNF-α on extracellular H2O2 production in HAECs. HAECs were pretreated with various concentrations of TNF-α (10, 20, 100, or 200 ng/mL) for (a) 30 mins, (b) 1 h, (c) 2 h, (d) 4 h, and (e) 6 h. The supernatant was collected, and the assay was performed immediately using H2O2 assay kits. The results are presented as the mean ± SEM from three independent experiments (n = 3). #P < 0.05 as compared to the unstimulated control. Supplementary Figure 3. Effect of H2O2 on SOD activity in HAECs. HAECs were pretreated with various concentrations of H2O2 (50, 100, 200, and 400 μM) for (a) 30 mins, (b) 2 h, and (c) 4 h. Then, the cells were lysed in iced-cold buffer. The cell lysates were collected, and the assay was performed using SOD assay kits. Data are presented as the mean ± S.E.M of three independent experiments (n = 3). #P < 0.05 as compared to the unstimulated control. Supplementary Figure 4. Effect of H2O2 on CAT activity in HAECs. HAECs were pretreated with various concentrations of H2O2 (50, 100, 200, and 500 μM) for (a) 30 mins, (b) 2 h, and (c) 4 h. Cell lysates were collected, and CAT activity was measured using CAT assay kits. Data are presented as the mean ± SEM of three independent experiments (n = 3). #P < 0.05 as compared to the unstimulated control. Supplementary Figure 5: MS/MS spectrum of peaks 1–5. [file 8738435.f1.docx]

**Supplementary Figure 1:** **The time response** **of TNF-α on ROS levels.** HAEC were stimulated with 10 ng/mL of TNF-α for different durations (30 mins – 24 h). After staining the cells with H_2_-DCFDA for 30 mins, the relative fluorescence unit of each well was measured. Data are presented as the mean ± S.E.M of three independent experiments (n=3). **P*<0.005 compared to unstimulated control.

a)

b)

c)

d)

e)

**Supplementary Figure 2: The effect of TNF-α on extracellular H_2_O_2_ production in HAEC.** HAEC were pre-treated with various concentrations of TNF-α (10, 20, 100 or 200 ng/mL) for **a) 30 m, b) 1 h, c) 2 h, d) 4 h and e) 6 h**. The supernatant was collected and the assay was performed immediately using H_2_O_2_ assay kits. Results are presented as the mean ± S.E.M. from three independent experiments (n=3). #*P*<0.05 as compared to unstimulated control.

a)

b)

c)

**Supplementary Figure 3**: **The effect of H_2_O_2_** **on SOD activity in HAEC**. HAEC were pre-treated with various concentrations of H_2_O_2_ (50, 100, 200 and 400 µM) for **a) 30 m, b) 2 h and c) 4 h**. Then, the cells were lysed in cold buffer. Cell lysates were collected and the assay was performed using SOD assay kits. Data are presented as the mean ± S.E.M of three independent experiments (n=3). #*P*<0.05 as compared to unstimulated control.

a)

b)

c)

**Supplementary Figure 4**: **The effect of H_2_O_2_ on CAT activity in HAEC**. HAEC were pre-treated with various concentrations of H_2_O_2_ (50, 100, 200 and 500 µM) for **a) 30 m, b) 2 h and c) 4 h**. Cell lysates were collected and CAT activity was measured using CAT assay kits. Data are presented as the mean ± S.E.M of three independent experiments (n=3). #*P*<0.05 as compared to normal control.


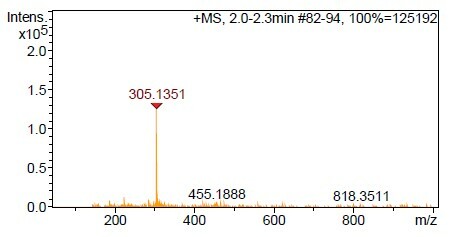


Peak 1

Peak 2


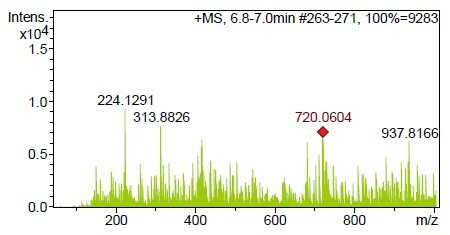


Peak 3


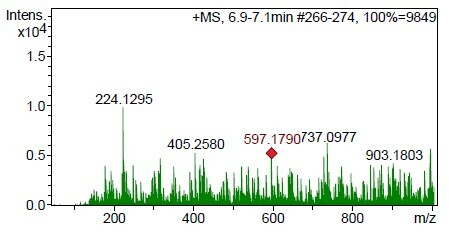


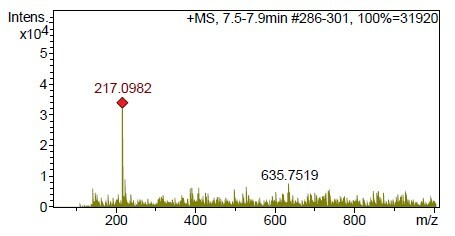


Peak 4

Peak 5


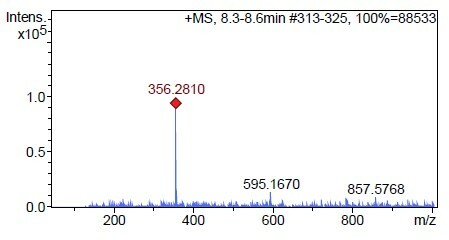


**Supplementary Figure 5: MS/MS spectrum of peaks 1-5.**
